# Unexpected abnormal flotation of gel separator in tube of post dialysis samples: a case report highlighting the critical role of sampling site selection

**DOI:** 10.11613/BM.2026.011001

**Published:** 2025-12-15

**Authors:** Anne Croisonnier, Vincent Bois, Guillaume Vernin, Carole Chirica, Dorra Guergour

**Affiliations:** 1 Immunoanalysis Biochemistry Unit, Department of Biochemistry, Molecular Biology and Environmental Toxicology, Institute of Biology and Pathology, Grenoble Alpes University Hospital, Grenoble, France; 2 AGDUC (Association Grenobloise des Dialysés et Urémiques Chroniques), Grenoble, France

**Keywords:** blood specimen collection, hemodiafiltration, renal dialysis, hyperproteinemia, preanalytical phase

## Abstract

Proper preanalytical handling of blood samples is critical to ensure the reliability of laboratory results, particularly in patients undergoing hemodialysis, where biochemical monitoring is essential for assessing dialysis adequacy and guiding treatment decision. We reported three cases of abnormal post-dialysis gel separator flotation in blood collection tubes from patients undergoing hemodiafiltration: in each case, the gel migrated to the top of the tube, with plasma trapped below and blood cells remaining at the bottom. Marked hyperproteinemia and hypercalcemia were observed in the plasma, inconsistent with the patient’s clinical status and pre-dialysis values. These findings raised suspicion of a preanalytical error potentially associated with the hemodialysis procedure. On-site investigations conducted in collaboration with the dialysis center for four additional patients, combined with a better understanding of the principles of hemodiafiltration and the potential sampling sites, confirmed that the gel migration anomaly was secondary to unsuitable sampling from the venous line (outflow line) of the dialysis circuit instead of the arterial one (inflow line). In conclusion, we highlighted the critical role of adhering to the appropriate sampling site when performing post-dialysis blood tests: the arterial line was identified as the appropriate site for post-dialysis blood sampling, while the venous line should be reserved exclusively for infusion or reinjection purposes and must never be used for blood collection at the end of dialysis.

## Graphical abstract

**Figure:**
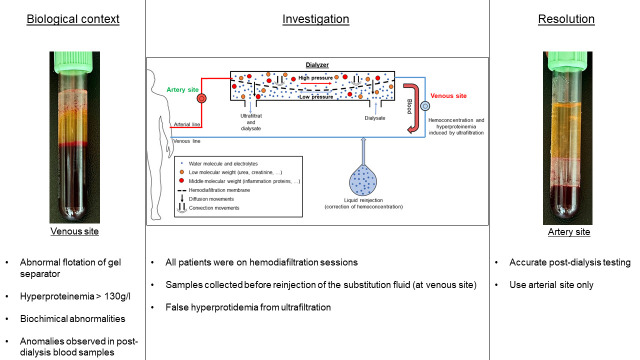

## Highlights

• Unexplained abnormal flotation of separator gel observed during hemodiafiltration

• Biochemical results discordant with the patient’s clinical condition

• The false-hyperproteinemia warrants evaluation for a preanalytical error

• Sampling from an inappropriate site was identified as the source of the anomaly

• The correct sampling site is essential to ensure valid hemodiafiltration results

## Introduction

Assessment of dialysis adequacy is a fundamental component of patient management in hemodialysis (HD). Among the most widely used indicators is the Kt/V index, which allows to assess dialysis adequacy. Urea measurement is required for the calculation of this index, where “K*”* represents urea clearance (L/min), “t*”* the duration of the dialysis session (min), and “V*”* the urea distribution volume (L). A target Kt/V value above 1.2 is generally recommended to ensure effective solute removal and optimize clinical outcomes (*1*). Monthly pre- and post-dialysis blood tests are therefore essential to ensure reliable Kt/V calculation and to prevent misinterpretation that could compromise patient care (*2*). Additionally, they provide essential information for monitoring electrolyte balance through the measurement of sodium, potassium, chloride, calcium, and protein concentrations, as well as for maintaining acid-base homeostasis by assessing bicarbonate (*3*). Rigorous control of preanalytical variables is crucial to ensure the reliability of laboratory results and to support optimal clinical decision-making (*4*). In our laboratory, blood samples from dialysis patients are collected using lithium heparin tubes with a gel separator. The separator gel is an inert, thixotropic polymer that alters its viscosity during centrifugation without interacting with blood components. Due to its specific density, intermediate between plasma and packed red blood cells, the gel typically forms a stable barrier between these two layers after centrifugation (*5*). These tubes offer several advantages: they eliminate the need for specimen aliquoting, provide a larger volume of usable plasma post-centrifugation, and minimize the risk of contamination from the cellular fraction due to the presence of the gel barrier. Moreover, samples collected in gel-containing tubes are more stable, ensuring proper preservation conditions of the specimens (*6*). Although infrequent, abnormal gel flotation due to an increase in plasma density may be observed. In cases such as hyperproteinemia or following administration of a contrast dye, analytical errors may result (*7*, *8*). We reported an unexpected flotation of the separator gel in blood samples from three patients, observed exclusively after hemodialysis. This anomaly was associated with discrepancies between pre- and post-dialysis laboratory results: marked, unexplained hyperproteinemia and severe hypercalcemia were detected post-dialysis, inconsistent with pre-dialysis values and the patients’ asymptomatic clinical status. This case report aimed to identify the potential causes of abnormal gel migration following centrifugation, as well as the aberrant biochemical values observed in post-dialysis blood samples.

**Figure 2:**
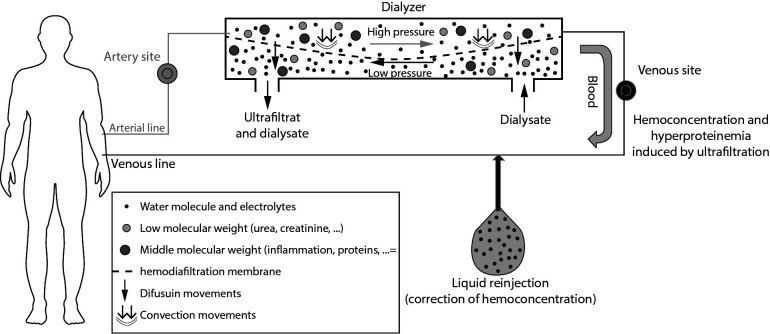
Principle of hemodiafiltration. Schematic representation of the extracorporeal circuit used during hemodialysis. Blood flows from the patient’s vascular access through the arterial line (inflow) to the dialyzer and returns *via* the venous line (outflow).

The three patients were undergoing hemodiafiltration (HDF) suggesting a dialysis-related issue that warrants further investigation. Conventional HD uses diffusion and convection across a semi-permeable membrane to facilitate renal clearance of waste products. Hemodiafiltration, in contrast, enhances the convection mechanism by applying positive pressure to the blood compartment, increasing filtration and promoting the elimination of medium molecular weight uremic toxins, including inflammatory proteins (*9*, *10*). However, this process also results in a significant loss of water and plasma electrolytes, necessitating reinfusion of an isovolumic substitution fluid to compensate for these hydro electrolytic losses before returning the purified blood to the patient (Figure 2*).* We therefore hypothesized that blood sampling performed at the end of dialysis, before the reinfusion of the replacement fluid, could explain the observed hyperproteinemia, due to hemoconcentration induced by hemodiafiltration. The objective of our study was to demonstrate that the anomalies of gel migration and discrepancies between pre- and post-dialysis samples originate from a previously undescribed preanalytical error related to the sampling site, and to identify the responsible non-compliant sampling site observed.

This analysis was conducted as case report, involving data from patients. The treatment has been carried out in Grenoble University Hospital according to French current regulation about case report publication and after consultation with our data protection officer. All subjects were informed about the processing of their data and their rights and have all signed a case report consent form in accordance with legislation and the institutional requirements. The raw data supporting the conclusions of this article will be made available by the authors within respect of General Data Protection Regulation, without undue reservation.

## Laboratory analyses

We reported the case of an 89-year-old woman undergoing HDF for the management of end-stage renal disease, secondary to embolic thrombosis of the renal arteries. Blood samples were collected at the initiation and conclusion of a dialysis session for routine biochemical analysis, including electrolytes, total protein, calcium, phosphate, urea, uric acid, and creatinine. Samples were drawn into BD Vacutainer lithium heparin tubes with gel separators (Becton Dickinson, Franklin Lakes, USA). The specimens arrived at the laboratory within 30 minutes of collection and were processed immediately. Preanalytical handling was performed using our automated system Aptio Laboratory Automation System (Inpeco SA, Novazzano, Switzerland), distributed by Siemens Healthineers. Samples were centrifuged at 2500xg for 10 minutes at 22 °C and subsequently analyzed using our automated clinical chemistry platform Atellica Solution (Siemens Healthineers, Tarrytown, USA).

**Figure 1:**
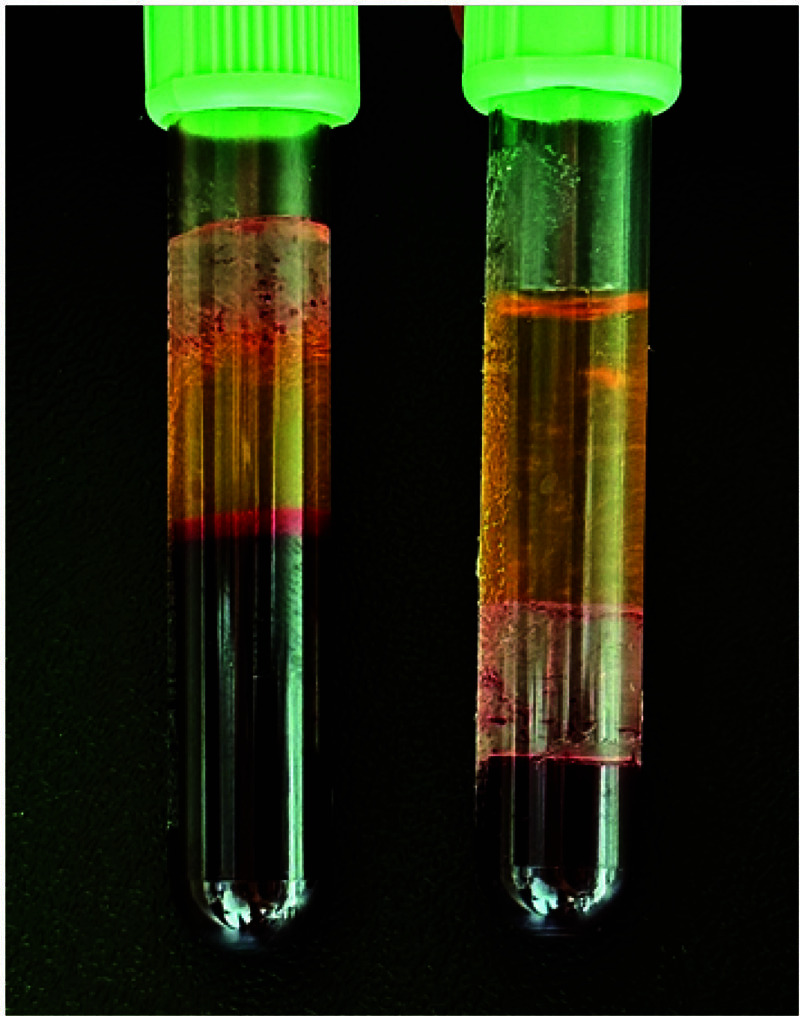
Lithium heparin plasma separator tubes following centrifugation. Left: abnormal flotation of the separator gel. Right: appropriate positioning of the separator gel following standard phase.

However, analysis of the post-dialysis sample was initially delayed due to an analyzer error related to the sample aspiration needle. Upon visual inspection, the laboratory technician identified an abnormal gel separation pattern in the post-dialysis tube (Figure 1, left tube): the separator gel had migrated to the topmost layer, while the plasma remained in the middle, and the packed blood cells at the bottom. After carefully recovering the plasma located beneath the separator gel, the prescribed post-dialysis biochemical analyses were performed. This issue recurred within the following month in two additional male patients, aged 75 and 77 years, undergoing HDF for diabetic nephropathy and idiopathic membranous glomerulonephritis, respectively. In all three cases, blood collection was performed by different nursing staff using different dialysis machines. Table 1 summarizes the biochemical results obtained from pre- and post-dialysis samples. All three patients exhibited marked hyperproteinemia in the post-dialysis samples, with unexplained elevations in total protein concentrations compared to pre-dialysis values. In addition to various electrolyte abnormalities, laboratory testing revealed severe hypercalcemia and hypokalemia, inconsistent with the patients’ asymptomatic clinical status. Blood urea nitrogen concentrations were undetectable, precluding the calculation of Kt/V. Following discussions with the treating physicians, and in light of these inconsistencies, none of the results from the affected samples were reported. Blood tests were repeated without urgency during the next dialysis session.

**Table 1:** Comparative analysis of biochemical parameters pre- and post-dialysis in patients exhibiting abnormal separator gel flotation in post-dialysis blood samples.

	**Case 1**		**Case 2**		**Case 3**	
**Parameter**	**Pre-dialysis**	**Post-dialysis**	**Pre-dialysis**	**Post-dialysis**	**Pre-dialysis**	**Post-dialysis**
Sodium (mmol/L)	144	133	133	127	137	130
Potassium (mmol/L)	4.1	2.6	3.5	2.0	4.9	3.0
Chloride (mmol/L)	110	91	95	80	101	77
Bicarbonate (mmol/L)	26	26	23	27	21	26
Calcium (mmol/L)	2.1	3.8	1.9	3.8	2.2	3.3
Phosphate (mmol/L)	1.2	< 0.1	1.4	< 0.1	2.0	< 0.1
Total protein (g/L)	62.0	149.4	79.0	168.1	74.0	169.4
Creatinine (µmol/L)	295	47	443	77	799	159
Uric acid (mmol/L)	331	34	462	ND	386	45
Urea (mmol/L)	15.3	< 1.8	28.0	< 1.8	19.3	< 1.8
ND - Not determined.						

## Further investigations

To investigate potential sampling errors, a collaboration was established with the dialysis center (AGDUC – Association Grenobloise des Dialysés et Urémiques Chroniques). Following discussions with the medical team, we attempted to reproduce the gel migration anomaly associated with hyperproteinemia and hypercalcemia in four additional patients, distinct from those previously described, all of whom were undergoing hemodiafiltration. Pre-dialysis samples were collected from the arterial site. Post-dialysis blood tests were performed in these four patients using two distinct sampling sites on the extracorporeal circuit: (*1*) the arterial line upstream of the dialyzer, and (*2*) the venous line downstream of the dialyzer but upstream of the substitution fluid reinfusion port (Figure 2).

Results are presented as mean ± 95% confidence interval (CI) for each parameter in the pre-dialysis, post-dialysis venous, and post-dialysis arterial groups. Using Stata version 18.0 (College Station, Texas, USA), the mean difference between post-dialysis venous and arterial samples, along with its 95% CI, was computed accordingly.

## What happened?

Following centrifugation of blood samples collected from both arterial and venous sites, a clear anomaly in the migration of the separator gel was consistently observed in tubes drawn from the venous site in all four patients. In contrast, samples collected from the arterial site exhibited normal gel migration, with the gel properly situated between the plasma and the pellet, as expected. Table 2 summarizes the biochemical results for each defined group. Venous blood samples demonstrated biochemical abnormalities consistent with our three initial cases, including marked hyperproteinemia (mean 147.3 g/L, 95% CI: 112.1 to 182.4) and severe hypercalcemia (mean 3.6 mmol/L, 95% CI: 3.2 to 3.9). The mean difference in plasma total protein was 70.5 g/L, with a 95% CI of 35.4 to 105.6 g/L, and the mean difference in plasma calcium was 1.1 mmol/L, with a 95% CI of 0.6 to 1.6 mmol/L. For all outcomes, the 95% CIs of the mean differences between the two sampling sites excluded zero, thus providing an argument for the presence of a signal for a difference.

**Table 2:** Comparative study of biochemical parameters in predialysis and postdialysis according to the sampling site

**Parameter**	**Blood sampling**			
	**Pre-dialysis** **(arterial A)** **(N = 4)**	**Post-dialysis** **(arterial A)** **(N = 4)**	**Post-dialysis** **(venous V)** **(N = 4)**	**Post-dialysis** **(V-A)** **(N = 4)**
Sodium (mmol/L)	138 (133 to 143)	137 (134 to 139)	131 (126 to 137)	- 5 (- 9 to - 1)
Potassium (mmol/L)	5.0 (4.3 to 5.6)	3.5 (3.0 to 4.0)	2.3 (0.9 to 3.7)	- 1.2 (- 2.2 to - 0.1)
Chloride (mmol/L)	102.1 (99.3 to 104.9)	97.5 (94.2 to 100.9)	86.3 (75.8 to 96.7)	- 11.3 (- 18.8 to - 3.8)
Calcium (mmol/L)	2.2 (1.9 to 2.6)	2.5 (2.2 to 2.7)	3.6 (3.2 to 3.9)	1.1 (0.6 to 1.6)
Phosphate (mmol/L)	1.7 (0.2 to 3.1)	0.6 (0.3 to 0.9)	0.1* (0.1 to 0.1)	- 0.5 (- 0.8 to - 0.2)
Total protein (g/L)	70.6 (58.9 to 82.2)	76.8 (61.7 to 91.9)	147.3 (112.1 to 182.4)	70.5 (35.4 to 105.6)
Creatinine (µmol/L)	644.5 (458.3 to 834.3)	228.8 (161.1 to 293.4)	77.5 (65.9 to 90.4)	- 151.3 (- 245.9 to - 56.6)
Urea (mmol/L)	19.9 (16.2 to 23.6)	6.8 (2.0 to 3.6)	1.8* (1.8 to 1.8)	- 5.0 (- 8.1 to - 1.8)
*All measured values were below the lower threshold of detection. For statistical purpose the minimum value of detection was assigned. Data are expressed as mean (95% confidence intervals). The mean difference between post-dialysis venous and arterial samples was estimated with its 95% confidence interval.				

## Discussion

The results of our investigations confirm that the observed protein migration anomaly and hyperproteinemia were indeed of preanalytical origin: post-dialysis blood samples in our patients were collected from the venous line instead of the arterial line. A better understanding of the principles of HDF allowed us to hypothesize that the hyperproteinemia was secondary to hemoconcentration induced by ultrafiltration. This hemoconcentration was evident in the post-dialysis samples, notably in the sample from the left-hand tube (Figure 1), which showed abnormally enlarged red blood cells, consistent with elevated hematocrit values. The observed hyperproteinemia provided a plausible explanation for the flotation anomaly, as previously described by Fatás *et al.* in patients with multiple myeloma (*11*). Furthermore, Faught *et al*. observed abnormal flotation of the separator gel in clinical samples with total protein concentrations above 162 g/L in BD collection tubes (*12*). As described in our case report, Demir *et al.* also reported abnormal flotation of separator gel in blood collection tubes used in HD patients, with marked increases in protein concentrations exceeding 168 g/L in post-dialysis samples (*13*). However, in this study, upon reviewing medical records, it was found that all three patients exhibiting flotation anomalies were hypotensive following the dialysis session. The authors concluded that hyperproteinemia was due to hemoconcentration caused by hypovolemia. In contrast, our patients were entirely asymptomatic. Our study presents compelling evidence in favor of hemoconcentration and hyperproteinemia being of preanalytical origin, related to post-dialysis blood sampling from the venous (outflow) line instead of the arterial (inflow) line. The venous site is clearly identified as an inappropriate site for post-dialysis sampling in patients undergoing hemodiafiltration. Our findings could be strengthened by conducting a study with a larger patient cohort to achieve methodological conclusiveness. However, the benefit of including additional patients to obtain statistically significant results appears limited, particularly as the sampling method for pre- and post-dialysis urea measurement is clearly defined in the KDOQI guidelines: samples must be taken from the arterial line (*2*). Our case underscores the importance of strictly adhering to these recommendations and identifies three major risks. First, the Kt/V cannot be interpreted from samples drawn from the venous site, as the measured urea concentration falls below the quantification threshold. Alternatively, dialysis dose can be estimated using the urea reduction ratio (URR), calculated as: (pre-dialysis urea - post-dialysis urea) / pre-dialysis urea (*14*). The minimum recommended dialysis dose corresponds to a URR of 60%. In our four patients, the risk of overestimating the URR is easily understood if calculated using a detection threshold of 1.8 mmol/L. The same applies to the calculation of Kt/V. Second, the electrolyte disturbances observed in post-dialysis samples drawn from the venous site underscore the risk of inappropriate management of hypokalemia or hypercalcemia if these results were reported. Third, aspiration of the gel, when it is not properly positioned in the tube, may occur during sample processing by laboratory instrumentation, especially in highly automated laboratories where primary collection tubes are centrifuged and analyzed without direct visual inspection by personnel. The instrument’s needle can become partially or completely obstructed by the gel, potentially compromising the integrity of the device. Needle replacement may be required, which could lead to delays in result turnaround time.

In conclusion, our case highlights the critical importance of using the appropriate sampling site for post-dialysis blood testing, as well as the potential risks associated with not adhering to established guidelines. The arterial line was identified as the correct site for post-dialysis sample collection, whereas the venous line should be strictly reserved for infusion or reinjection purposes and must not be used for blood collection at the end of dialysis.

## What you can do in your laboratory to prevent such errors

Continuous education of clinical teams on adherence to correct blood sampling protocols and their influence on laboratory results is essential. The critical risk associated by improper sampling site selection, especially for post-dialysis tubes, and its subsequent impact must be comprehensively managed by healthcare personnel.

Clinical laboratories must provide healthcare teams of the AGDUC with informational leaflets containing detailed and specific instructions addressing all aspects of the preanalytical phase.

Communication between the clinical laboratory and clinical departments must be strengthened.

If a non-conformity is identified, it should be documented and communicated to the prescribing physician in the results report. This practice will facilitate the assessment of deviations from sampling protocols and improve nurse training where necessary.

## Data Availability

All data genered and analyzed in the presented study are included in this published article.

## References

[1] Canaud B Fouque D. Recommandations européennes de bonnes pratiques (EBPG) en hémodialyse. Deuxième vague [European recommendations for good practice in hemodialysis. Part Two]. Nephrol Ther. 2008;4:115–24. 10.1016/j.nephro.2007.12.002 18326481

[2] National Kidney Foundation. KDOQI Clinical Practice Guideline for Hemodialysis Adequacy: 2015 update. Am J Kidney Dis. 2015;66:884–930. 10.1053/j.ajkd.2015.07.015 26498416

[3] Nodimar C Chauveau P. Argumentaire justifiant le dosage des marqueurs biochimiques de routine en hémodialyse [Argument justifying the measurement of routine biochemical markers in hemodialysis]. Nephrol Ther. 2024;20:143–6. 10.1684/ndt.2024.74 38742304

[4] Gendt L Szymanowicz A. Proposition pour la maîtrise de la phase pré-analytique selon la norme NF EN ISO 15189. Bio Trib Mag. 2010;36:50–8. 10.1007/s11834-010-0022-8

[5] Lima-Oliveira G Lippi G Salvagno GL Gelati M Bassi A Contro A Abnormal gel flotation caused by contrast media during adrenal vein sampling. Biochem Med (Zagreb). 2016;26:444–50. 10.11613/BM.2016.047 27812311 PMC5082217

[6] Parra-Robert M Rico-Santana N Alcaraz-Quiles J Sandalinas S Fernández E Falcón I Improvement in the stability of serum samples stored in an automated refrigerated module. Clin Biochem. 2016;49:1396–8. 10.1016/j.clinbiochem.2016.10.012 27789213

[7] van den Ouweland JMW Church S. High total protein impairs appropriate gel barrier formation in BD Vacutainer blood collection tubes. Clin Chem. 2007;53:364–5. 10.1373/clinchem.2006.081653 17259253

[8] Daves M Lippi G Cosio G Raffagnini A Peer E Dangella A An unusual case of a primary blood collection tube with floating separator gel. J Clin Lab Anal. 2012;26:246–7. 10.1002/jcla.21512 22811356 PMC6807415

[9] Hannedouche T Bachelet T Roy FL Canaud B. Place de l’hémodiafiltration en ligne dans le traitement de suppléance rénale de l’insuffisance rénale chronique ultime en 2022 : situation actuelle et perspectives. [Place of on-line hemodiafiltration in renal replacement therapy for chronic end-stage renal disease in 2021: Current status and perspectives]. Nephrol Ther. 2022;17:3S5-17/3S11. 10.1016/S1769-7255(22)00033-5

[10] Canaud B Chénine L Leray-Moraguès H Patrier L Rodriguez A Gontier-Picard A Hémodiafiltration en ligne : modalités pratiques, sécurité et efficacité de la méthode [Online hemodiafiltration: Practical aspects, safety and efficacy]. Nephrol Ther. 2017;13:189–201. 10.1016/j.nephro.2017.02.007 28483384

[11] Fatás M Franquelo P Franquelo R. Anomalous flotation of separator gel: density or viscosity? Clin Chem. 2008;54:771–2. 10.1373/clinchem.2007.093716 18375495

[12] Faught RC Marshall J Bornhorst J. Solution densities and estimated total protein contents associated with inappropriate flotation of separator gel in different blood collection tubes. Arch Pathol Lab Med. 2011;135:1081–4. 10.5858/2010-0488-OAR.1 21877989

[13] Demir M Ozlem S Sarier M. Abnormal Flotation of Separator Gel in Blood Test Tubes in the Hemodialysis Patients. EJMI. 2019;3:280–4. 10.14744/ejmi.2019.82037

[14] Schortgen F. Tolérance et efficacité des séances d’épuration extrarénale [Tolerance and efficacy of renal replacement therapy]. Reanimation. 2003;12:318–26. 10.1016/S1624-0693(03)00062-8

